# Synergistic potential of Berberine and Wogonin improved adipose inflammation and insulin resistance associated with obesity through HIF-α axis

**DOI:** 10.1186/s13020-025-01223-w

**Published:** 2025-10-03

**Authors:** Zihan Zhou, Mingsu Wang, Xiaoyu Zhang, Yinyue Xu, Ping Li, Zuguo Zheng, Hua Yang

**Affiliations:** State Key Laboratory of Natural Medicines, China Pharmaceutical University, Nanjing, 211198 China

**Keywords:** Berberine, Wogonin, Insulin resistance, Obesity, Synergize, HIF-α

## Abstract

**Background:**

Obesity-induced adipose hypoxia activates the hypoxia-inducible factor alpha (HIF-α) axis, where HIF-1α and HIF-2α exhibit functional antagonism: HIF-1α exacerbates insulin resistance (IR) and inflammation, whereas HIF-2α counteracts these effects. This dichotomy suggests dual targeting (inhibiting HIF-1α/activating HIF-2α) could synergistically ameliorate obesity-associated IR. Coptidis Rhizoma and Scutellariae Radix, a classic traditional Chinese medicine (TCM) pair for damp-heat obesity-demonstrate efficacy, but their synergistic components and mechanistic basis remain unclear. Therefore, this study aims to identify active ingredients through the dual targeting strategy and subsequently validate their therapeutic efficacy.

**Methods:**

Based on the dual-targeting strategy, potential active ingredients were identified using a specifically designed dual-luciferase reporter system that simultaneously inhibits HIF-1α and activates HIF-2α. The optimal synergistic ratio was determined by applying the Loewe model. In vitro anti-inflammatory efficacy was evaluated in macrophages stimulated with palmitic acid or lipopolysaccharide. In vivo effects were assessed in a high-fat diet-induced obese mouse. Insulin sensitivity was determined by measuring fasting blood glucose levels and performing oral glucose tolerance test and insulin tolerance tests. Tissues were analysed for lipid metabolism and inflammatory markers via immunohistochemistry, quantitative real-time PCR, and enzyme-linked immunosorbent assay.

**Results:**

Berberine (BBR) and wogonin (WOG) were identified from herb pair to inhibit HIF-1α and activate HIF-2α in an optimal synergistic ratio of 3:1. At this ratio, the combination significantly ameliorated the secretion of pro-inflammatory cytokines in vitro. Consistent with the in vitro findings, co-administration of BBR and WOG (3:1) showed lipid-lowering effects comparable to metformin and effectively improved insulin sensitivity in obese mice. Additionally, improved lipid metabolism-related parameters including plasma total cholesterol and free fatty acids, thereby mitigating hepatic lipid accumulation.

**Conclusions:**

The synergistic constituents of Coptidis Rhizoma-Scutellariae Radix, namely BBR and WOG, synergistically alleviate IR associated with obesity by inhibiting HIF-1α and activating HIF-2α, respectively. This study elucidates the mechanistic basis of herbal combination therapy for metabolic disorders and provides a foundation for developing novel synergistic therapeutics against obesity-related IR.

**Supplementary Information:**

The online version contains supplementary material available at 10.1186/s13020-025-01223-w.

## Introduction

Obesity is a metabolic disorder characterized by excessive adipose tissue expansion due to prolonged energy intake exceeding expenditure, representing a major global public health concern [1]. The etiology of obesity is complex, with substantial evidence associating it with persistent, low-grade systemic inflammation [2–4]. This inflammation is implicated in the pathogenesis of metabolic disorders, including insulin resistance (IR), type 2 diabetes mellitus (T2DM), and cardiovascular diseases [5]. IR, the most prevalent obesity-related disease, is characterized by reduced insulin sensitivity. This manifests clinically as diminished glucose uptake and energy expenditure compared to healthy individuals, contributing to the development of impaired adipose tissue function, dyslipidemia, and inflammation [6, 7]. Consequently, chronic adipose tissue inflammation is recognised as a key driver in the development of obesity-related metabolic dysfunction.

Recent research progress underscores the pivotal role of adipose tissue hypoxia in inflammation. Hypoxia arises from insufficient oxygen supply to the target tissue and is orchestrated by hypoxia-inducible factors (HIFs), which act as core transcription factors sensing cellular oxygen levels and mediating adaptive responses [8, 9]. HIFs are heterodimers composed of alpha and beta subunits. The oxygen-dependent alpha subunit includes two well-characterized isoforms, HIF-1α and HIF-2α, each possessing distinct functions and target genes [10]. Crucially, they exert opposing biological effects within hypoxic adipose tissue. HIF-1α has been shown to upregulate inducible nitric oxide synthase (iNOS) transcription, leading to nitric oxide (NO) production. This stimulates inflammation via reactive oxygen species (ROS), impairs adipocyte function, and exacerbates obesity and IR [11]. Conversely, HIF-2α maintains NO homeostasis by reducing NO generation and promoting arginase-1 (ARG1) expression, which competes with iNOS for the substrate L-arginine. Failure of HIF-2α to exert its inhibitory function allows persistent HIF-1α activation, accelerating the inflammatory response in obesity and contributing to IR development [11]. Based on this functional antagonism, we propose that dual-targeting inhibition of HIF-1α alongside activation of HIF-2α may synergistically ameliorate obesity-induced inflammation and IR.

Traditional Chinese medicine (TCM) possesses a long-standing therapeutic tradition for managing obesity and diabetes. According to TCM theory, obesity stems from internal physiological imbalances, such as damp-phlegm accumulation and spleen-stomach deficiency [12, 13]. Disease progression typically manifests pathological states including phlegm-turbidity, damp-heat, and blood stasis [13]. Herbal pairs represent empirically validated, fixed-ratio combinations of two medicinal herbs, established through systematic clinical practice to achieve synergistic therapeutic effects [14]. These dual-herb formulations are commonly administered in combination to enhance clinical efficacy, target specific pathophysiological manifestations, or mitigate potential adverse effects [15, 16]. The Coptidis Rhizoma-Scutellariae Radix herbal pair, characterised by its bitter-cold properties and capacity to clear damp-heat, constitutes a clinically recommended therapeutic strategy for obesity associated with damp-heat internal retention syndrome [16, 17]. Scutellariae Radix (the dry rhizome of *Scutellaria baicalensis* Georgi), first recorded in the Shennong Herbal Classic, contains predominant bioactive flavonoids including baicalein, wogonin (WOG), and oroxylin A as its major constituents [17–19]. Modern pharmacological studies have demonstrated the anti-obesity, anti-dyslipidemic, and anti-hyperglycemic effects of the major flavonoids in Scutellariae Radix in rodent models, highlighting their therapeutic relevance for metabolic disorders [17, 20]. Coptidis Rhizoma (the dry rhizome from *Coptis chinensis* Franch, *Coptis deltoidea* C.Y.Cheng et Hsiao or *Coptis teeta* Wall.), a widely used clinical herb, contains alkaloids as its principal active ingredients, particularly berberine (BBR), coptisine, and palmatine [17, 21, 22]. Multiple studies have documented the lipid-lowering, lipogenesis-inhibition, and anti-obesity effects of CR-derived alkaloids [16, 23]. Notably, BBR has been mechanistically validated to ameliorate hepatic steatosis via suppression of the LPS/TLR-4 pathway, while concurrently exerting anti-inflammatory effects and reducing lipid synthesis and accumulation in adipocytes [24, 25]. Emerging evidence indicates that the Scutellariae Radix-Coptidis Rhizoma herb pair exerts therapeutic effects in T2DM rats by modulating the MAPK/PI3K/AKT pathway, improving glucose homeostasis and lipid metabolism while attenuating chronic inflammation [17, 26, 27]. This dual-action mechanism suggests potential benefits in obesity prevention and delaying diabetic complications. However, the comprehensive therapeutic profile of this herb pair in addressing obesity-related metabolic disorders remains limited by an incomplete understanding of its specific synergistic components and underlying mechanisms.

Current therapies for obesity or T2DM predominantly rely on long-term medication (e.g., metformin, glipizide, and pioglitazone) or invasive bariatric surgery, both of which are associated with significant adverse effects and impose substantial financial burdens on patients. In contrast, current pharmacological research has confirmed the precise efficacy and favourable safety profile of Coptidis Rhizoma-Scutellariae Radix treatment. This study aims to elucidate the role of BBR and WOG-synergistic bioactive constituents screened from the classic herbal pair Coptidis Rhizoma-Scutellariae Radix-in ameliorating obesity-linked IR through modulation of HIF-α isoforms. The findings contribute to advancing the pharmacological understanding of herbal pairs, facilitating their ongoing development, and establishing a foundation for novel therapeutic strategies targeting obesity-associated IR.

## Materials and methods

### Reagents

The 28 major chemical components in Coptidis Rhizoma-Scutellariae Radix were commercially available, all purchased from Chengdu Must Biotechnology and Chengdu Prufa Technology Development Co., Ltd, and the purity were above 98% (Table 2). HIF-1α KO cell (No. RM01984) was purchased from Wuhan Aibotec Biotechnology Co. Ltd. Bovine insulin powder (No. BS901) was purchased from Biosharp Corporation, USA. Palmitic acid (PA) (No. P0500) was purchased from Sigma Corporation, USA. COCl_2_, NC_000006.12 in pGL3-basic (ARG1) and NC_000017.11 in pGL3-basic (iNOS) were purchased from Suzhou Jinweizhi Biotechnology Co., Ltd. lipopolysaccharide (LPS) (No. BS007) and phosphate-buffered saline (PBS) powder (No. BL601A) were purchased from Biosharp. DMEM (No. KGL1206-500) was received from Key GEN BioTECH (Nanjing, China). South American fetal bovine serum (No. 10270-106), 0.25% trypsin EDTA (No. 25200072) and Opti mem serum reducing medium were purchased from GIBCO from United States. Lip3000 transfection reagent was purchased from Thermo Fisher Scientific. Ampicillin (No. HY-B0522A) and Kanamycin sulfate (No. HY-16566A) were purchased from MCE. DH5α competent cells (No. 9057) were purchased from Takara Corporation of Japan. Plasmid extraction kit (No. AP-MN-P-250) was purchased from Axygen (USA). β-galactosidase reporter gene detection kit (No. RG0036), total nitric oxide assay kit (No. S0021S) and CCK-8 cell-counting kit (No. C0038) were purchased from Shanghai Biyuntian Biotechnology Co., Ltd. Reporter lysis 5 × buffer (E3971) and luciferase assay substrate (E151A) were purchased from Promega (USA). Mouse interleukin 1β (IL-1β; No. CSB-E08054m), IL-6 (No. CSB-E04539m), tumor necrosis factor-α (TNF-α; No. CSB-E04741m), monocyte chemoattractant protein-1 (MCP-1; No. CSB-E07430m), insulin (No. CSB-E05071m), resistin (No. CSB-E06886m), leptin (No. CSB-E04650m), and adiponectin (No. CSB-E07272m) ELISA kits were purchased from Elabscience (Wuhan, China). Total cholesterol (TC) (No. A111-1-1), triglyceride (TG) (No. A110-1-1), low-density lipoprotein-cholesterol (LDL-C) (No. A113-1-1), high-density lipoprotein-cholesterol (HDL-C) (No. A112-1-1), free fatty acids (FFA) (No. A042-2-1), lactate (No. A019-2-1), phenylalanine aminotransferase (No. C009-2-1), and aspartate aminotransferase (No. C010-2-1) assay ELISA kits were purchased from Jiancheng Bioengineering Institute (Nanjing, China). Anti-CD11b (No. AF300251), anti-CD11c (No. AF300249), anti-F4/80 (No. AFW18637), anti-HIF-1α (No. AF300536) and HIF-2α (No. AF300154) were purchased from Hunan AiFang Biotechnology Co., Ltd. Ripa lysis buffer (Cat. No. P0013B), 5 × loading buffer (Cat. No. P0015L), and BCA protein assay kot (Cat. No. P0010) were purchased from Beyotime (Shanghai, China). Anti-HIF-1α antibody (Cat. No. 36169S) and anti-HIF1-2α antibody (Cat. No. 59973S) were purchased from Cell Signaling Technology (CST, MA, USA). β-actin antibody (Cat. No. 20536-1-AP), anti-ARG1 antibody (Cat. No. 16001-1-AP), anti-iNOS antibody (Cat. No. 22226-1-AP) and HRP-conjugated Goat Anti-Rabbit IgG (H + L) (Cat. No. SA00001-2) were purchased from Proteintech (Wuhan, China).

### Cell culture and treatment

RAW264.7 macrophage, 3T3-L1 preadipocyte and HEK-293T were obtained from the American Type Culture Collection (ATCC; TIB-71, CL-173™ and CRL-3216™, respectively). RAW264.7 cells and HEK-293T cells were cultured in DMEM high glucose medium supplemented with 10% fetal bovine serum (FBS; Gibco) and 1% (v/v) penicillin–streptomycin under standard conditions (37 °C, 5% CO_2_). 3T3-L1 cells were cultured in DMEM supplemented with 10% newborn calf serum (NBCS; Gibco) and 1% (v/v) penicillin–streptomycin at 37 °C under 5% CO_2_ and were passed at a ratio of 1:2. RAW264.7 and HEK-293 T cells were passaged at a ratio of 1:3. A 20 mM stock solution of each drug in dimethyl sulfoxide (DMSO; Sigma, No. D2650;) was prepared based on their respective molar masses. The stock solutions were diluted in cell culture medium to a working concentration of 20 μM (final DMSO concentration = 0.1%). A 10 mM PA solution was prepared by dissolving 53.5 mg of PA in 0.1 mM NaOH, followed by heating in a 70 °C water bath until clarity was achieved. A 4% bovine serum albumin (BSA) solution was then rapidly added to adjust the final PA concentration to 5 mM, and the solution was stored at − 80 °C. A 1 mg/ml LPS stock solution was prepared by dissolving 1 mg LPS powder in 1 mL PBS, followed by vortexing and centrifugation to ensure complete dissolution. The LPS solution was stored at − 80 °C.

### Determination of NO content

Following induction with LPS and PA, the cell supernatant was collected by centrifugation at 4 °C. A standard curve was generated using serially diluted standard with concentrations of 0, 1, 2, 5, 10, 20, 40, 60, and 100 μM. Subsequently, 50 μL of Griess reagent I and 50 μL of Griess Reagent II were added to each well and thoroughly mixed. Absorbance was measured at 450 nm using an enzyme-linked immunosorbent assay (ELISA) reader (BioTek, USA). The concentration of nitric oxide (NO) in the samples was then calculated based on the standard curve.

### CCK-8 cell viability assay

RAW264.7 cells were seeded in 96-well plates of 1 × 10^4^ cells per well. After cells had reached approximately 70% confluence, they were co-treated with different concentration of BBR or WOG, along with 100 μM PA, for 24 h. Subsequently, the CCK-8 reagent was added to each well at a 1:100 dilution in culture medium, thoroughly mixed, and incubated at 37 °C in a 5% CO_2_ atmosphere for 30 min. Absorbance at 450 nm was measured using an enzymatic spectrophotometer (BioTek, USA) following the incubation periods.

### Cell transfection

Plasmids were extracted using a standard kit. Cells in the logarithmic growth phase were harvested, seeded into 96-well plates (Thermo, USA), and transfected upon reaching 70–80% confluence. For bacterial transformation, competent cells and plasmids were kept on ice. A 2 μL aliquot of plasmid was added to the competent cells and mixed gently. The mixture was then incubated on ice for 30 min, subjected to a 42 °C heat shock for 60 s in a water bath, and immediately returned to ice for 2 min. Subsequently, the mixture was transferred to a 37 °C incubator and shaken at 200 rpm for 2 h. A 200 μL aliquot of the transformed bacterial culture was plated onto selective solid culture medium containing the containing the appropriate antibiotics and incubated at 37 °C for 12 h. Following plasmid extraction, the bacterial culture was centrifuged at 12,000 r/min for 1 min, and the supernatant was discarded. The pellet was resuspended in 350 μL of Solution S1 and transferred to a 1.5 mL sterile centrifuge tube. Subsequently, 250 μL of Solutions S2 and S3 were added, followed by 8 inversions for mixing. The mixture was centrifuged at 12,000 r/min for 10 min. After centrifugation of the supernatant at 12,000 r/min for 1 min, the lower phase was discarded. Elution was performed with 500 μL of Solution W1, and two washes with Solution W2 were conducted. Then, 60 μL of eluent buffer was added. Following a 5 min incubation, centrifugation at 12,000 r/min for 1 min was performed, and the lower phase was collected, followed by sequential addition of Lipofectamine 3000 reagent and the target plasmid, with thorough mixing to ensure homogeneity. Post-transfection, cells were incubated for 24 h before medium replacement to sustain cultivation.

### Dual luciferase reporter system

Following 24 h of cell transfection, COCl_2_ was utilised for modelling and drug administration [28]. The control group was treated with 3% FBS-DMEM medium, while the model group was exposed to 200 μM COCl_2_. The drug-treated groups were administered with individual drug mixture (20 μM each) plus 100 μM COCl_2_. After an additional 24 h incubation, cells were washed twice with PBS, and the reporter gene lysate was diluted to 1 × concentration. The lysate was transferred to a clean white opaque 96-well plate, followed by addition of the luciferase substrate under light-protected conditions. Reporter fluorescence intensity was detected at 450 nm using a microplate reader. Absorbance was measured with a β-Gal detection kit and normalised against luciferase activity via the dual luciferase reporter system.

### Quantitative real-time PCR

Total RNA was extracted from RAW264.7 cells, 3T3-L1 cells, and epididymal white adipose tissue (eWAT) using TRIzol reagent (R701; Vazyme, Nanjing, China). For mice tissues, approximately 65 mg of eWAT was minced into pre-beaded centrifuge tube. After addition of 500 μL RNA-easy lysis buffer, tissues were homogenized for 15 min at low temperature for subsequent gene expression analysis. RNA concentration was quantified with an ultra-micro spectrophotometer (AoSheng, China). cDNA was synthesized by reverse transcription using 5 × HiScript II qRT SuperMix II (R223-01; Vazyme, Nanjing, China). qPCR was performed with SYBR Green Master Mix (Q421-02; Vazyme, Nanjing, China), and the cycle threshold (Ct) values were determined using a Light Cycler 480 qPCR System (Eppendorf, Germen) following standardised protocols. Target gene expression was normalized to GAPDH or β -action as internal references. qPCR primers were supplied by Kingsley Biotechnology Ltd, shown in Table 1. GraphPad Prism 8.0 was used for statistical analysis.

**Table 1:** Primer gene sequence

Gene	Forward (5′ to 3′)	Reverse (5′ to 3′)
IL-6	GCTACCAAACTGGATATAATCAGGA	CCAGGTAGCTATGGTACTCCAGAA
IL-1β	GTGCTGCCTAATGTCCCCTTGAATC	TGCAGAGTTCCCCAACTGGTACATC
β-actin	GGCTGTATTCCCCTCCATCG	CCAGTTGGTAACAATGCCATG
ARG1	GCTTCGGAACTCAACGGGAGGG	ACCAGAAAGGAACTGCTGGGATACA
MCP-1	TACCTTTTCCACAACCACCTC	ATTAAGGCATCACAGTCCGAGT
CCL2	CATCCACGTGTTGGCTCA	GATCATCTTGCTGGTGAATGAGT
F4/80	CCCAGTGTCCTTACAGAGTG	GTGCCCAGAGTGGATGTCT
CD11b	ATGGACGCTGATGGCAATACC	TCCCCATTCACGTCTCCCA
TNFα	CCCTCACACTCAGATCATCTTCT	GCTACGACGTGGGCTACAG
IL-10	GCTCTTACTGACTGGCATGAG	CGCAGCTCTAGGAGCATGTG
TGF-β1	CTCCCGTGGCTTCTAGTGC	GCCTTAGTTTGGACAGGATCTG
HIF-1α	ACCTTCATCGGAAACTCCAAAG	CTGTTAGGCTGGGAAAAGTTAGG
HIF-2α	CTGAGGAAGGAGAAATCCCGT	TGTGTCCGAAGGAAGCTGATG
iNOS	CTGCAGCACTTGGATCAGGA	TGGTGAGGGAGTGGTGTAG

### Analysis of synergistic anti-inflammatory effects

Based on the Loewe additivity model, the theoretical framework proposed by Chou and Talalay was applied [29, 30]. The drug’s dose–effect relationship was assumed to conform to: log[E/(1 − E)] = α (log*d *− logD_m_), which constitutes a linear regression model with log[E/(1 − E)] as the effect index and log*d* as the regresoor. This model was employed for fixed-ratio conminations of BBR and WOG. Here, E represents the NO inhibition rate, d denotes drug dose, D_m_ indicates the median-effect dose (i.e., IC_50_), and α is the slope constant. Median-effect plots were generated from this regression model based on individual drug NO inhibition rates. For a given effect X produced by a drug NO inhibition rates. For a given effect X produced by a drug combinayion (*d*_BBR_, *d*_WOG_), the dose required for BBR alone (D_X, BBR_) and WOG alone (D_X, WOG_) were detived from the median-effect plot. The interaction index τ was defined as: τ = *d*_BBR_/D_X, BBR_ + *d*_WOG_/D_X, WOG._

### In vitro model of synergistic effects on macrophages

The synergistic effects of BBR and WOG in macrophages were investigated in vitro. RAW264.7 cells were seeded in 6-well or 24-well plates until 80% confluency was reached. Cells were then treated with BBR (0, 1.25, 2.5, and 5 μM) or WOG (0, 1.25, 2.5, and 5 μM), and co-cultured with 100 μM PA for 24 h or 1 μg/mL LPS for 12 h [31–33]. Cell supernatant were collected and centrifuged to assess concentration-dependent effects of individual drug treatments. Subsequently, a combination of 3.75 μM BBR plus 1.25 μM WOG (3:1 ratio) was administered to cells, with comparisons made against 5 μM BBR or 5 μM WOG monotherapy groups. Anti-inflammatory effects on macrophages were quantifies using an ELISA kit.

### ELISA

Cell supernatant was collected from LPS- and PA-stimulated cultures into 1.5 mL centrifuge tubes. Samples were subsequently centrifuged at 4 °C and 1000 × g for 15 min, followed by careful collection of the supernatant. Assay reagents were equilibrated at room temperature (18–25 °C) for at least 30 min and prepared according to the manufacturer’s instructions. Standard and test sample wells were established separately with 100 μL of standard or test sample added to each well. Expression levels of inflammatory factors (IL-1β, IL-6, TNF-α) in RAW264.7 cells supernatant, along with concentrations of MCP-1, insulin, resistin, leptin, lipocalin, aspartate aminotransferase (AST), and alanine aminotransferase (ALT), were quantified using corresponding ELISA kits per manufacturer’s protocols.

### Western blot analysis

Proteins from 3T3-L1 cells were lysed with RIPA buffer (containing protease and phosphatase inhibitors; Roche, Switzerland). The protein concentrations were quantified with a BCA assay kit, followed by mixing with 5 × loading buffer. Samples were denatured at 100 °C for 10 min, separated by SDS-PAGE, and transferred to PVDF membranes. Membranes were blocked with 5% (w/v) skin milk in TBST for 1 h, then incubated overnight at 4 °C with primary antibodies (1:1000 dilution) against: HIF-1α, HIF-2α, iNOS, ARG1 and β-actin. After washing, membranes were incubated with HRP-conjugated secondary conditions (Beyotime, China) for 1 h at room temperature (25 °C). Protein signals were detected using a Tanon 5200 Chemiluminescent Imaging System.

### Animal studies

Male C57BL/6J mice, (7–8 weeks old, SPF grade, 20–22 g) were housed at the Laboratory Animal Centre of China Pharmaceutical University (CPU, Nanjing, China) under controlled conditions: 12 h light/dark cycle, 23 ± 3 °C, and 55 ± 15% relative humidity. The experimental protocol was approved by the Institutional Animal Ethics Committee (Approval No. 2021–12-010). After a 1-week acclimatization period, mice were randomly allocated to six groups: control diet (ND), high-fate fiet (HFD), metformin (Met) (200 mg/kg), BBR (40 mg/kg) [34], WOG (40 mg/kg) [35], and BBR + WOG (3:1, 40 mg/kg) [36]. BBR, WOG and metformin were dissolved in saline. All mice were administered via daily oral gavage, with the HFD and ND groups receiving equivalent volumes of saline. Except for the ND group fed standard chow (∼10% fat, Xietong, Nanjing, China), all other groups were maintained on a high-fat diet (∼60% fat, D12492, Research Diets, USA) for 14 weeks. Food was withheld for 12 h prior to euthanasia. Blood was collected via orbital bleeding and centrifuged at 3,000 rpm for 15 min. Serum was then assayed for t total cholesterol (TC), triglyceride (TG), high-density lipoprotein (HDL-C), low-density lipoprotein cholesterol (LDL-C), and free fatty acid (FFA) using commercial kits. Serum levels of insulin, resistin, leptin, lipocalin, AST, ALT, IL-1β, IL-6, and MCP-1 were quantified by ELISA with a double-antibody sandwich method. Following cervical dislocation, adipose tissue samples (epididymal white adipose tissue, eWAT; perinephric white adipose tissue, pWAT; subcutaneous white adipose tissue, sWAT; inguinal white adipose tissue, iWAT; and brown adipose tissue, BAT) and livers were harvested, weighed, flash-frozen in liquid nitrogen, and stored at − 80 °C. The acute toxicity test was also measured. A single oral gavage of 400 mg/kg BBR-WOG (3:1) suspension (10 × clinical dose) was administered to mice [37, 38]. The control group received 0.9% saline solution. After 7 days, brain, heart, liver, spleen, lungs, kidneys and muscle tissues were collected for H&E staining.

### Metabolic analysis

At week 14, basal metabolism in mice was examined. The 24-h metabolic data were acquired using the TSE PhenoMaster V5.8.6 Metabolic Assay System (CLAMS; TSE PhenoMaster, Thuringia, Germany) following a 24-h acclimatization period and in accordance with manufacturer’s instructions. Oxygen consumption (V_O2_), CO_2_ release (V_CO2_), energy expenditure (EE), and respiratory exchange rate (RER) were calculated over a 24 h period [39].

### Fasting blood glucose (FBG), oral glucose tolerance (OGTT), and insulin tolerance (ITT) measurements in mice

FBG measurements were performed during week 10 of the modelling period. Mice were subjected to a 12-h fast prior to the procedure [40]. A small incision (1–2 mm) was created at the tail tip, and the first drop of blood discarded. Subsequently, the blood glucose meter, equipped with pre-inserted test strips, was brought into contact with blood droplet for absorption, and the reading was displayed. For the OGTT, mice were fasted for 12 h and administered a 0.2 g/mL dextrose solution via oral gavage at a constant dose. Blood glucose levels were measured using a glucometer at 15, 30, 60, 90, and 120 min. For the ITT, mice were fasted for 5 h and administered 0.05 U/kg insulin solution via intraperitoneal injection at a constant rate, with blood glucose levels measured at the aforementioned time points.

### Immunohistochemical staining

Immediately following euthanasia, eWAT and the liver specimens were fixed in 4% paraformaldehyde overnight and subsequently embedded in paraffin. Paraffin sections of the liver (5 mm) were mounted onto slides for oil red staining; paraffin sections of eWAT (5 mm) were subjected to hematoxylin and eosin staining (H&E) staining and immunohistochemical staining for F4/80 (1:100), CD11b (1:100), CD11c (1:100), HIF-1α (1:100), and HIF-2α (1:100) (all antibodies from AiFang biological, Hunan, China). After drying, the neutral gum-monted sections were observed and photographed using an inverted fluorescence microscope (Ts2R; Nikon, Tokyo, Japan).

### Data statistics and analysis

Statistical analysis was performed using GraphPad Prism 8.0, and the data were expressed as mean ± SD or mean ± SEM (n ≥ 3). Comparisons among multiple groups were conducted using one-way ANOVA, and comparisons between two groups were conducted using Student’s *t*-test. Statistical significance was defined as follows: *P < 0.05, **P < 0.01, ***P < 0.001; results with *P* ≥ 0.05 were considered non-significant (ns).

## Results

### Establishment of dual luciferase reporter system for synergistic component identification in coptidis rhizoma—scutellariae radix

**Fig. 1:**
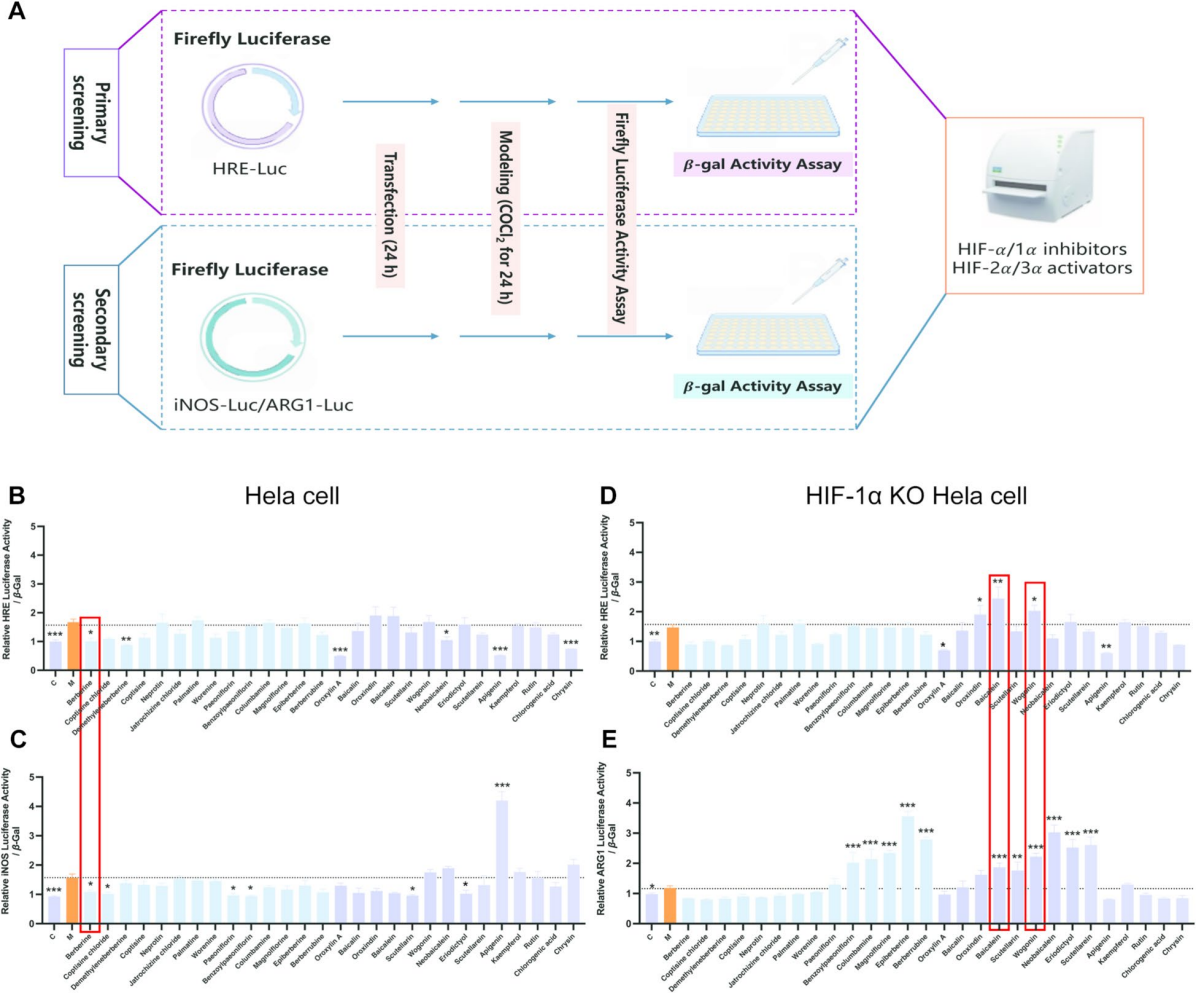
Screening active components in Coptidis Rhizoma-Scutellariae Radix based on HIF-axis. **A** Flowchart of the luciferase reporter gene system: the luciferase reporter gene was constructed and transfected into Hela cells or HIF-1α knockout cells. COCl_2_ was used to simulate hypoxia, and different drug stimuli were applied. The fluorescence value and β-Gal activity in cell lysates were measured. **B** Construction of the HRE-Luc luciferase reporter gene was used to screen for active ingredients inhibiting HIF-α, n = 3. **C** Construction of an iNOS-Luc luciferase reporter gene to screen for active ingredients that inhibit HIF-1α, n = 3. **D** The HRE-Luc luciferase reporter gene was constructed to screen the active components of HIF-2α/3α agonists (HIF-1α^−/−^ cells), n = 3. **E** Construction of ARG1-Luc luciferase reporter gene to screen for active components of HIF-2α agonists (HIF-1α.^−/−^ cells), n = 3. (***P < 0.001, **P < 0.01, *P < 0.05, *ns* not significantly different)

A high-throughput cellular screening system utilizing a dual luciferase reporter was constructed (Fig. 1A). Briefly, the HRE-Luc reporter plasmid, containing a hypoxia response element, was transiently transfected into either Hela cells or HIF-1α knockout cells to select HIF-α inhibitors or HIF-2α activators, respectively, under COCl_2_-induced hypoxia conditions. The iNOS-Luc promoter luciferase reporter plasmid was then transfected into Hela cells to evaluate HIF-1α inhibition. Similarly, the ARG1-Luc promoter reporter plasmid was transfected into HIF-1α knockout cells to screen for HIF-2α activators under hypoxia. Meanwhile, β-Gal was co-transfected as an internal reference reporter gene for data normalisation. Following compound stimulation, luciferase activity and β-Gal activity in cell lysates were measured, and the ratio was utilized to determine compounds efficacy. Based on phytochemical evidence, the primary active constituents for metabolism disorders therapy in Coptidis Rhizoma are alkaloids [41–44], while in Scutellariae Radix they are flavonoids (Table 2) [45]. Therefore, these two classes of active compounds were primarily selected for efficacy screening. Utilising this approach, transfection of the constructed HRE-Luc and iNOS-Luc promoter reporter genes (targets of HIF-1α) into Hela cells demonstrated inhibitory activity by berberine (BBR) (Fig. 1B, C). Similarly, transfection of the HIF-2α target gene ARG1-Luc reporter plasmid into HIF-1α knockout (KO) cells identified baicalein and wogonin (WOG) as specific HIF-2α agonists (Fig. 1D, E), which activated both HRE-Luc and ARG1-Luc simultaneously. To further validate HIF-2α agonism, the ARG1 Luc-luciferase system was assessed without COCl_2_-induced hypoxia. Under both normoxic and hypoxic conditions, only WOG significantly enhanced ARG1-Luc transcriptional activity (Fig. S1A). Subsequently, the effects of baicalein and WOG on the gene expression of ARG1 and downstream NO levels in LPS/PA-induced RAW264.7 inflammation models were investigated [33, 46]. CCK8 results indicate that the optimal concentration for PA modelling is 100 μM, and LPS is 1 μg/mL (Fig. S1B, C). While both compounds reduced NO elevation (Fig. S1D, E), only WOG significantly reversed both LPS and PA-induced suppression of ARG1 mRNA (Fig. S1F, G). Baicalein showed no significant effect on ARG1 gene expression in the PA-induced model. These results establish WOG as the optimal HIF-2α agonist. Together, considering the efficacy against HIF-1α and HIF-2α target genes (*i.e.*, iNOS and ARG1), BBR and WOG were selected for further evaluation of synergistic efficacy due to their superior effectiveness.

**Table 2:** Main active ingredients in coptidis rhizoma and scutellariae radix

	Compound	Category	Origin	References
1	Berberine	Alkaloids	Coptidis rhizoma	[41]
2	Coptisine chloride			[42]
3	Demethyleneberberine			[41]
4	Coptisine			[41]
5	Neprotin			[41]
6	Jatrochizine chloride			[43]
7	Palmatine			[41]
8	Worenine			[43]
9	Paeoniflorin			[44]
10	Benzoylpaeoniflorin			[44]
11	Columbamine			[41]
12	Magnolflorine			[41]
13	Epiberberine			[41]
14	Berberrubine			[43]
15	Oroxylin A	Flavonoids	Scutellariae radix	[45]
16	Baicalin			[45]
17	Oroxindin			[45]
18	Baicalein			[45]
19	Scutellarin			[45]
20	Wogonin			[45]
21	Neobaicalein			[45]
22	Eriodictyol			[45]
23	Scutellarein			[45]
24	Apigenin			[45]
25	Kaempferol			[45]
26	Rutin			[45]
27	Chlorogenic acid			[45]
28	Chrysin			[45]

**Fig. 2:**
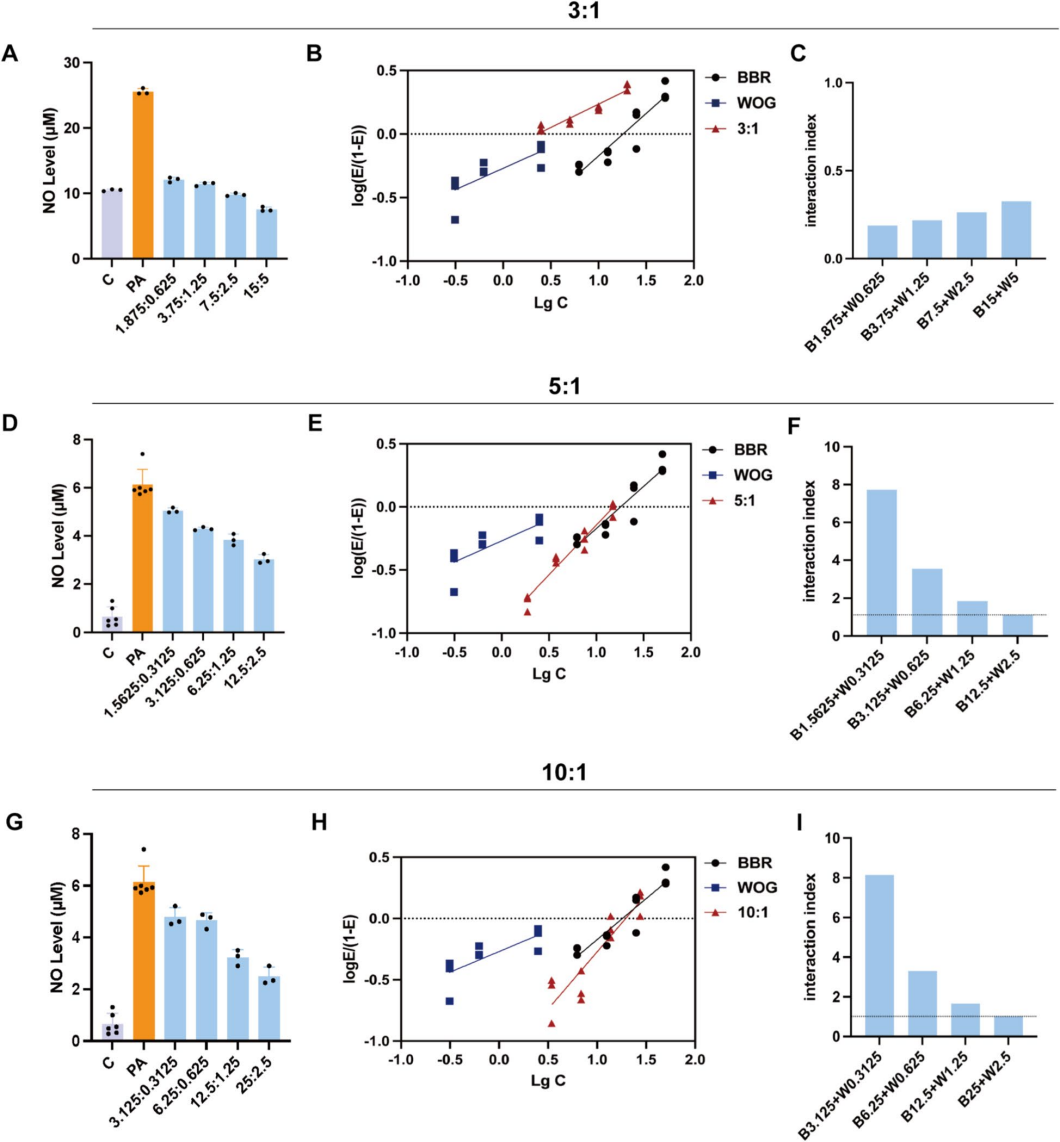
Synergistic anti-inflammatory effects of BBR and WOG in three ratios. The NO levels in RAW264.7 were measured after treatment with 100 μM PA and different concentrations of BBR and WOG for 24 h. Obtain the IC_50_ values of BBR, WOG and their combination based on their respective NO inhibition rate curves. The median effect plot analysis was obtained by using log[E/(1 − E)] = α (log*d *− logD_m_), where D_m_ is the IC_50_. Then, based on the dose effect curves of log[E/(1 − E)] and log*d* of BBR and WOG in the median effect plot, the interaction index of each combination group at different compatibility ratios was calculated. **A**–**C** Synergistic anti-inflammatory effect analysis of BBR and WOG in a 3:1 ratio, n = 3, **A** NO inhibition rate curve. **B** Median effect plot. **C** Interaction index. **D**–**F** Synergistic anti-inflammatory effect analysis of BBR and WOG in a 5:1 ratio, n = 3, **D** NO inhibition rate curve. **E** Median effect plot. **F** Interaction index. **G**–**I** Synergistic anti-inflammatory effect analysis of BBR and WOG in a 10:1 ratio, n = 3. **G** NO inhibition rate curve. **H** Median effect plot. **I** Interaction index

Next, the appropriate concentration ranges for BBR and WOG were determined using CCK8 assays. BBR significantly inhibited RAW264.7 cell proliferation at 100 μM (Fig. S2A), while WOG significantly reduced viability starting at 10 μM (Fig. S2B). Consequently, optimal concentrations were identified as 0–50 μM for BBR and 0–5 μM for WOG. At these concentrations, BBR and WOG exhibited concentration-dependent inhibition of their respective target genes, ARG1 and iNOS, following PA modelling (Fig. S2C, D). Furthermore, concentration–response analyses established IC_50_ values of 16.56 μM (BBR) and 7.26 μM (WOG) for NO inhibition (Fig. S2E, F), yielding a potency ratio of BBR:WOG **≈** 2.3:1. Critically, this ratio demonstrates that a minimum of 2.3 BBR units are required to achieve equivalent NO inhibitory activity to 1 WOG unit. To accurately characterise drug interactions in combinations, doses must approximate this potency equivalence (≥ 2.3:1 ratio) to prevent dominance by either compound [47, 48]. Consequently, compatibility ratios of 3:1, 5:1, and 10:1were systematically evaluated for combined efficacy assessment (Fig. 2) [36]. Further median-effect analysis and interaction indices (τ) calculations confirmed synergistic anti-inflammatory effects at a 3:1 BBR:WOG ratio (Fig. 2A–C), with τ < 1 indicating synergy across tested concentrations. However, antagonism occurred at WOG concentrations below 2.5 μM within these higher ratios (5:1 and 10:1) (τ > 1). Due to this antagonism, these ratios were excluded, and the 3:1 BBR:WOG ratio was selected for further evaluation of synergistic effects. Subsequently, we employed 3T3-L1 adipocytes, induced hypoxia with 200 μM COCl_2_, and stimulated inflammation with 100 μM PA or 1 μg/mL LPS (Fig. S5) [49, 50]. Using Western blot and PCR analysis, we systematically investigated the regulatory effects of BBR and WOG (both individually and in 3:1 combination) on HIF-1α/HIF-2α protein and gene expression. Furthermore, to further elucidate the mechanism, we concurrently examined changes in protein and gene expression of iNOS and ARG1, downstream targets of HIF-1α/HIF-2α. Results demonstrated that under hypoxic conditions, both PA and LPS stimulation upregulated protein and gene expression of HIF-1α and its downstream target iNOS (Fig. S5A-H & S5Q-T). BBR monotherapy suppressed these upregulations, while the BBR-WOG combination exhibited more pronounced inhibitory effects. Similarly, WOG monotherapy and combination treatment significantly upregulated HIF-2α and its target gene ARG1, with enhanced efficacy observed in the combination group (Fig. S5I-P & S5U-X). These findings align with the principle of our dual-luciferase reporter gene screening system, thereby providing experimental validation for the proposed HIF axis-mediated regulatory mechanism.

### Improvement of macrophage inflammation by BBR-WOG combination

**Fig. 3:**
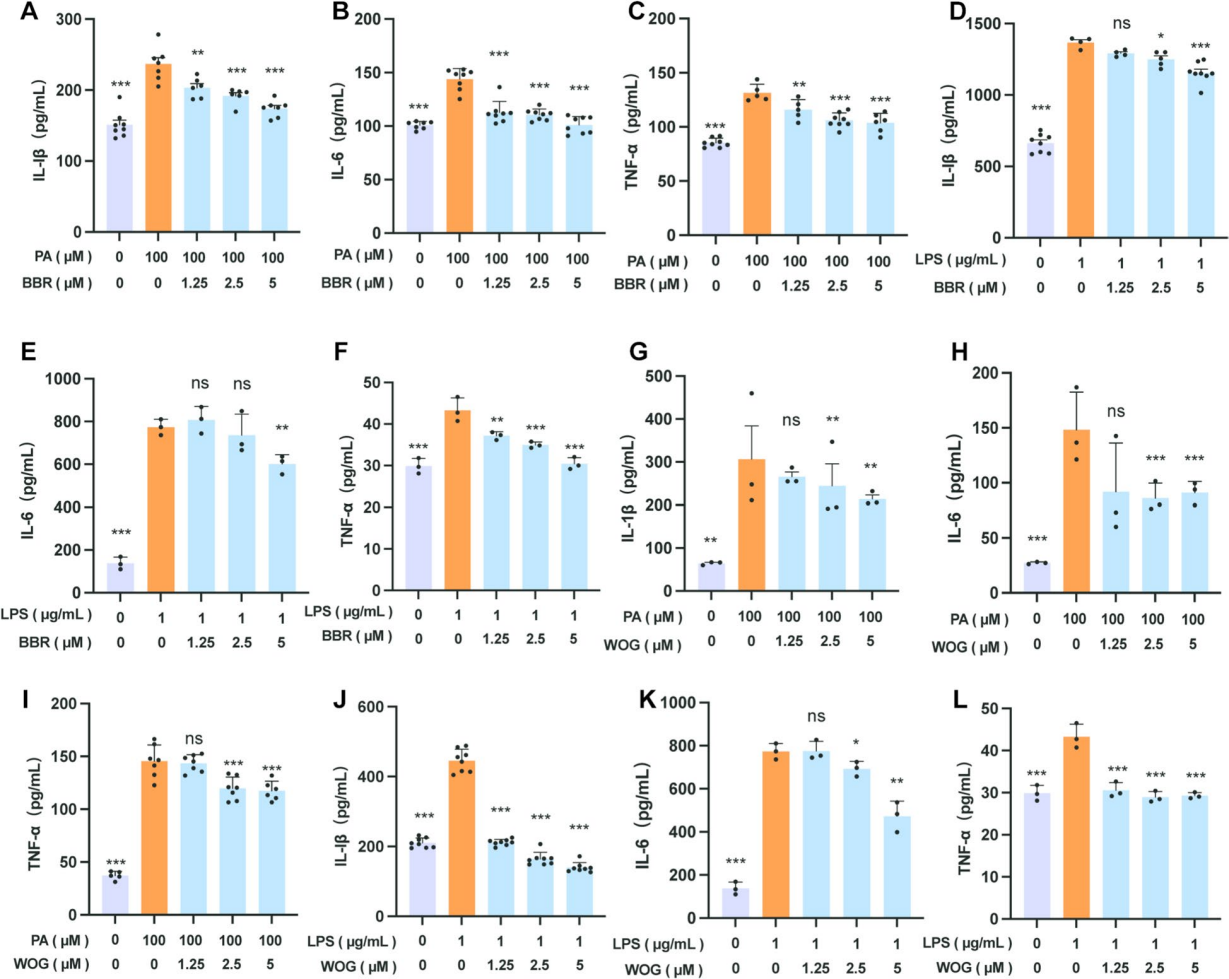
Ameliorative effects of BBR and WOG on macrophage inflammation. RAW264.7 was induced with 100 μM PA and 1 μg/mL LPS and treated with different concentrations of BBR and WOG for 24 h or 12 h. ELISA detected cytokine secretion in the cell supernatant. **A**–**F** Under PA and LPS modelling, the effects of BBR administration at concentration gradients of 1.25, 2.5, and 5 μM on the secretion of IL-6, IL-1β, and TNF-α, n ≥ 3. **G**-**L** Under PA and LPS modelling, the effects of WOG administration at concentration gradients of 1.25, 2.5, and 5 μM on the secretion of IL-6, IL-1β, and TNF-α, n ≥ 3. (***P < 0.001, **P < 0.01, *P < 0.05, *ns* not significantly different)

**Fig. 4:**
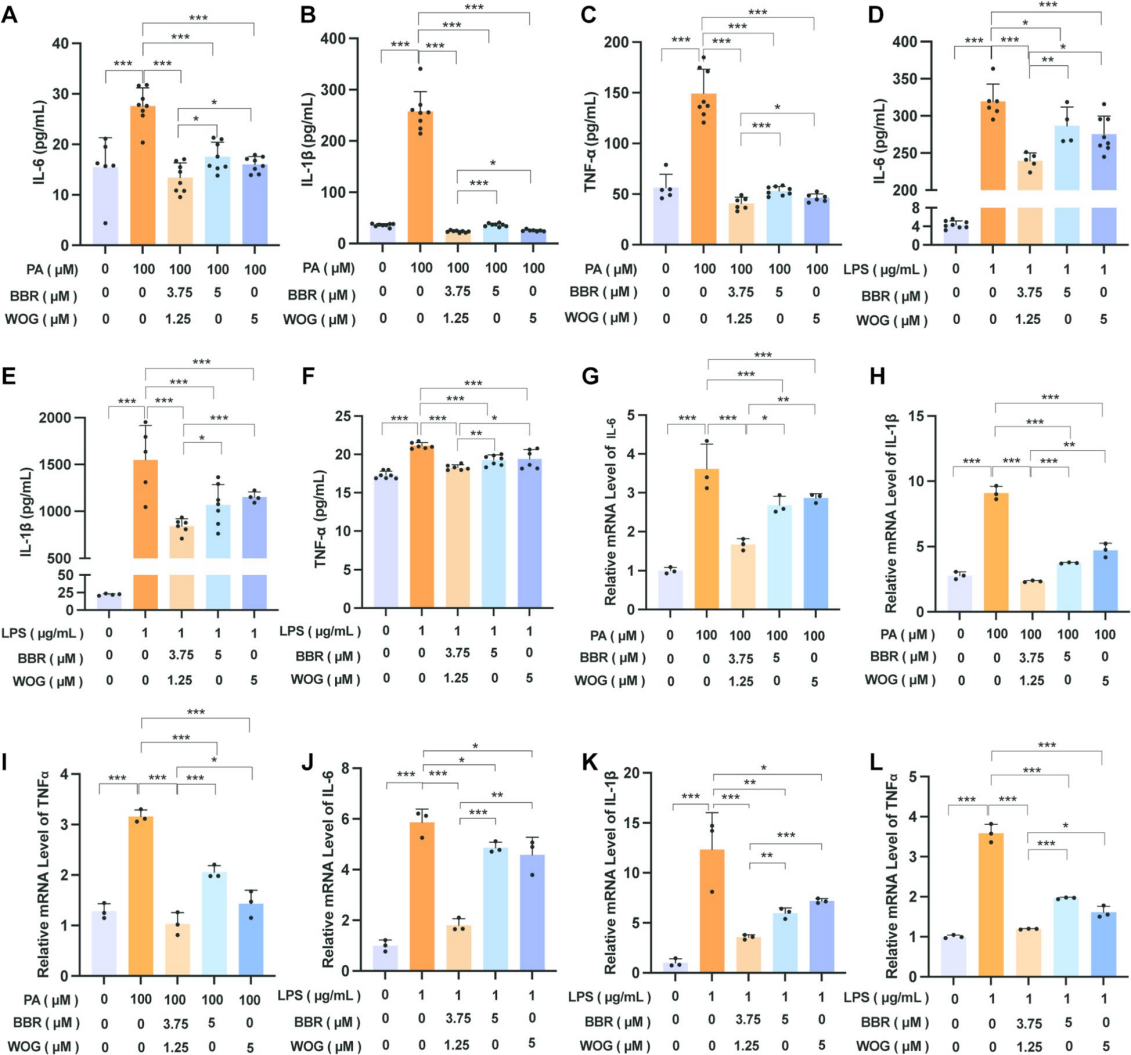
Ameliorative effects of BBR-WOG combination on macrophage inflammation. RAW264.7 was induced with 100 μM PA and 1 μg/mL LPS, and treated with BBR, WOG at 5 μM, or BBR: WOG 3:1 (3.75:1.25) for 24 or 12 h. ELISA detected cytokine secretion in cell supernatant. Gene expression was detected by qPCR. **A**-**F** The effects of BBR and WOG on the secretion of inflammatory factors IL-6, IL-1β, and TNF-α, respectively and in combination, under PA and LPS modelling conditions, n ≥ 3. **G**-**L** The effects of BBR and WOG, respectively and in combination, on the mRNA expression of IL-6, IL-1 β, and TNF-α under PA and LPS modelling conditions, n ≥ 3. (***P < 0.001, **P < 0.01, *P < 0.05, *ns* not significantly different)

Inflammatory cytokines play an essential role in the development of obesity-associated T2DM. Typically, the expression of HIF-1α in macrophages may trigger adverse physiological responses by regulating macrophage metabolic reprogramming and inflammation, leading to obesity and/or IR [51]. Therefore, RAW264.7 cells were primarily used to investigate the impact of BBR and WOG on inflammatory factor release and gene expression, as determined by ELISA and PCR. Results demonstrated that BBR significantly inhibited the secretion and gene expression of IL-6, IL-1β, and TNF-α under both PA and LPS stimulation (Fig. 3A–F and S3A–F). However, low concentrations (1.25–2.5 μM) of BBR failed to inhibit IL-6 secretion in LPS-induced supernatants, though efficacy was restored at 5 μM (Fig. 3E). In contrast, WOG exhibited minimal effects on inflammatory factor secretion during PA modelling but demonstrated significant inhibitory effects at 2.5 μM (Fig. 3G–I). Moreover, under LPS conditions, WOG dose-dependently inhibited TNF-α secretion and gene expression (Figs. 3L and S3L), as well as IL-6 and IL-1β (Figs. 3J, K and S3J, K). Notably, inhibition of IL-6 secretion was evident starting at 2.5 μM WOG. The efficacy of BBR-WOG combination therapy in mitigating macrophage inflammation was assessed relative to individual treatments. While treatment with BBR or WOG alone reduced the secretion and gene expression of IL-6, IL-1β, and TNF-α in both PA-(Figs. 3A–F and S3A–F) and LPS-induced (Figs. 3G–L and S3G–L) cells, a synergistic inhibitory effect was observed with the 3:1 BBR:WOG combination (Fig. 4). Compared with monotherapy (BBR or WOG alone at 5 μM each), the 3:1 combination of BBR and WOG (3.75 μM: 1.25 μM) significantly reduced the secretion levels (Fig. 4A–F) and gene expression levels (Fig. 4G–L) of key pro-inflammatory cytokines (e.g., TNF-α, IL-6, IL-1β) in both PA-induced and LPS-induced inflammation models. These results demonstrate that the anti-inflammatory efficacy of the 3:1 BBR:WOG combination is significantly superior to that of either agent administered alone. Furthermore, we evaluated the effects of BBR and WOG administered individually or in combination on the secretion levels and gene expression of the anti-inflammatory cytokines IL-10 and TGF-β1 (Fig. S4). Cell supernatant was collected following inflammatory modeling with PA and LPS, as well as subsequent drug treatment, and analyzed by ELISA. The results demonstrated that, compared to the individual treatment groups (BBR and WOG administered as 5 μM each), the 3:1 co-treatment group significantly reversed the suppression of IL-10 and TGF-β1 secretion induced by inflammatory modelling (Fig. S4E-H). Consistent with this, mRNA expression analysis performed under the same conditions revealed analogous effects (Fig. S4A–D). Collectively, the 3:1 BBR-WOG ratio effectively mitigated intracellular inflammatory responses induced by high-fat diets, highlighting its potential for ameliorating macrophage inflammation.

### Synergistic amelioration of insulin resistance in mice by BBR-WOG combination therapy

**Fig. 5:**
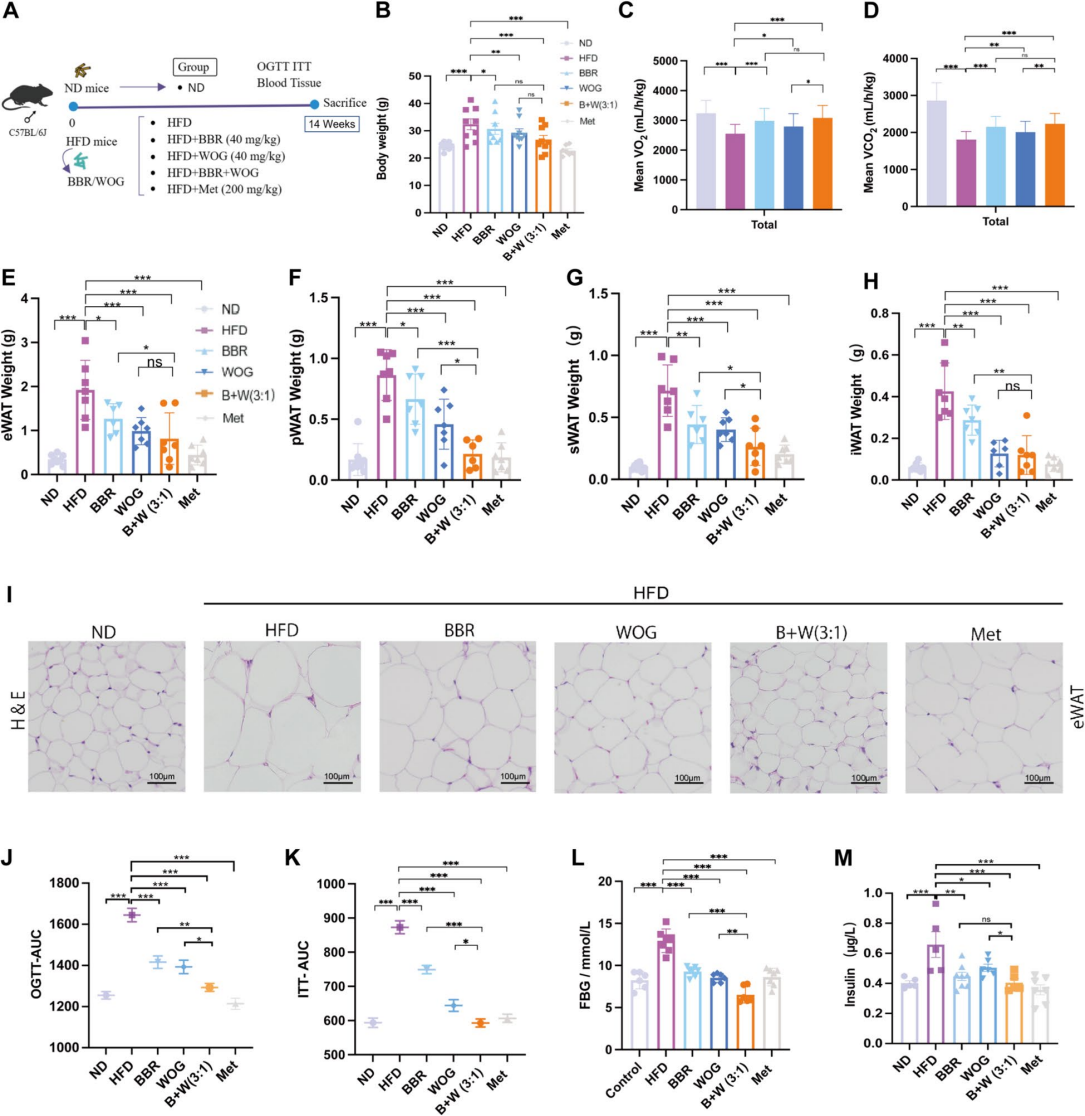
Effects of BBR-WOG combination on basal metabolism and IR in DIO mice. Mice were fed a high-fat diet and administered for 14 weeks, the Met group was given at 200 mg/kg and the other groups were given at 40 mg/kg dose rate. **A** Schematic diagram of the in vivo evaluation of drug efficacy. **B** Body weight of mice after fasting, n ≥ 6. **C** 24-h oxygen consumption, n = 3. **D** 24-h carbon dioxide release, n = 3. **E** Weight of eWAT, n ≥ 6. **F** Weight of pWAT, n ≥ 6. **G** Weight of sWAT, n ≥ 6. **H** Weight of iWAT, n ≥ 6. **I** H&E staining of mouse eWAT, 20×, 100 μm, n ≥ 6. **J** Area under curve of OGTT of 10-week DIO mice, n ≥ 6. **K** Area under curve of ITT for 10-week DIO mice, n ≥ 6. **L** FBG of 13-week DIO mice, n ≥ 6. **M** Fasting insulin level in serum of 14-week DIO mice, n ≥ 6. (***P < 0.001, **P < 0.01, *P < 0.05, *ns* not significantly different)

To investigate the efficacy of BBR and WOG in vivo, we employed a diet-induced obese (DIO) mouse model established by 14 weeks of high-fat diet feeding (Fig. 5A). Mice were randomised into the following groups: normal diet (ND), HFD model, treatment groups (BBR, WOG, BBR + WOG), and positive control (Met). Based on the clinically relevant dose of the Coptidis Rhizoma-Scutellariae Radix herb pair (6–9 g each, 1:1 ratio), the calculated equivalent dose of BBR in mice was approximately 40 mg/kg [52–54]. Given that both BBR and WOG exhibit significant bioactivities-including anti-diabetic, anti-inflammatory, and anti-tumour effects-at this dosage [55–58], we selected 40 mg/kg to optimally evaluate their synergistic potential. Concurrently, we conducted an acute toxicity assessment of the drug combination. Using tenfold the clinical dose, histopathological evaluation of major organs performed 7 days post single-administration revealed no significant toxicity (Fig. S6). Compared to ND controls, HFD-fed mice displayed significant weight gain, which was attenuated post-treatment. Notably, although ND mice consumed more food, their body weight remained lower than DIO mice, likely attributable to the higher energy content of HFD (Fig. S7A-C). The BBR + WOG (3:1) combination normalised food intake in DIO mice to ND levels (Fig. S7B). Additionally, metabolic analysis revealed reduced 24-h oxygen consumption (VO₂) and carbon dioxide production (VCO_2_) in HFD mice, which were restored upon treatment (Figs. 5C, D and S7D, E). Evaluations of the RER (Fig. S7H–J) and EE (Fig. S7K-M) further demonstrated significant improvements, particularly with the 3:1 BBR-WOG combination.

Investigation of adipose tissue (AT) weight in mice, particularly within white adipose tissue depots including eWAT (Fig. 5E), pWAT (Fig. 5F), sWAT (Fig. 5G), iWAT (Fig. 5H), revealed increased mass under HFD. This increase was significantly reduced following treatment. Notably, combined administration of BBR and WOG at a 3:1 ratio effectively reduced the weight of pWAT and sWAT, exhibiting efficacy comparable to Metformin (Met) in reducing white fat mass. Consistent trends were observed for eWAT and overall fat mass (Fig. S7N, O), corroborated by H&E staining (Fig. 5I). Concurrently, elevated OGTT and ITT area-under-the-curve values compared to ND controls indicated impaired glucose tolerance and insulin sensitivity (Figs. 5J, K and S7P, Q). These impairments were ameliorated post-treatment (Fig. S7R), paralleling improvements in FBG and insulin levels (Fig. 5L, M). Both individual and combined administration of BBR and WOG, particularly the 3:1 combination (BBR + WOG), mitigated DIO-associated metabolic abnormalities. The efficacy of the BBR + WOG combination was comparable to the clinical standard, Met. Collectively, these results demonstrate the synergistic potential of BBR and WOG in improving metabolic outcomes in DIO mice.

### Enhanced control of macrophage infiltration and chronic inflammation by BBR-WOG combination

**Fig. 6:**
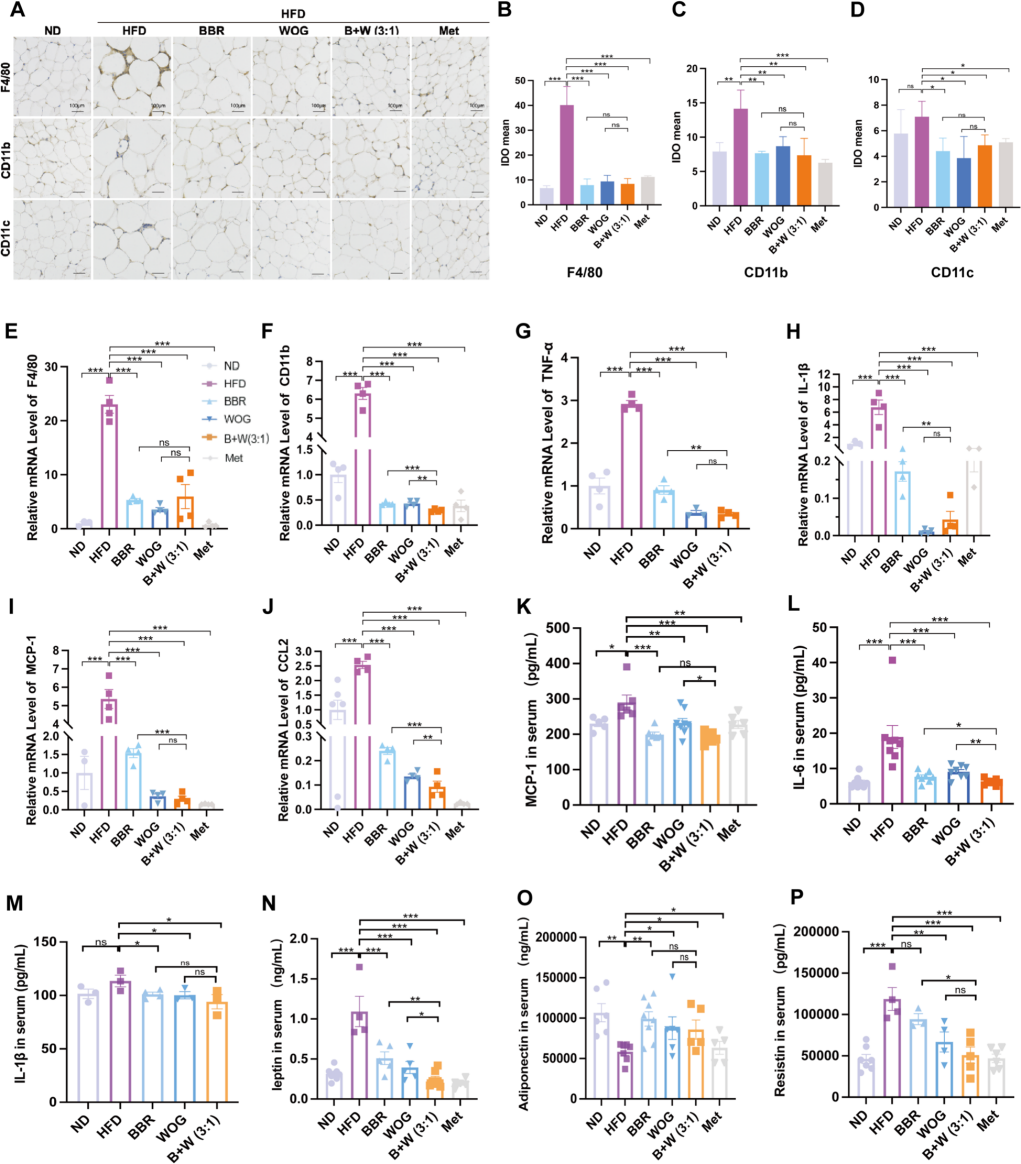
Effects of BBR-WOG combination on chronic inflammation in DIO mice. **A** Immunohistochemical evaluation of the changes in the levels of F4/80, CD11b and CD11c labelled ATMs in eWAT of DIO mice. **B**–**D** Quantitative statistical plots of (**A**), n = 3. **E**, **F** mRNA levels of macrophage marker genes Fe/80 and CD11b, n ≥ 3. **G**, **H** mRNA levels of inflammatory factors IL-β and TNF-α, n ≥ 3. **I**, **J** mRNAs levels of chemokines MCP-1 and CCL2, n ≥ 3. ELISA kits were used to measure serum levels of (**K-M**) MCP-1, IL-6 and IL-1β. (**N**-**P**) Leptin, lipocalin and resistin levels, n ≥ 6. (***P < 0.001, **P < 0.01, *P < 0.05, *ns* not significantly different)

Macrophage infiltration is a well-established phenomenon in white adipose tissue. Immunohistochemistry analysis of eWAT cross-sectional revealed a significantly higher proportion of F4/80^+^, CD11b^+^, and CD11c^+^ adipose tissue macrophages (ATMs; murine macrophage markers) in DIO mice compared to the ND group (Fig. 6A), indicating enhanced macrophage infiltration due to chronic HFD feeding. This infiltration was subsequently ameliorated following drug treatment (Fig. 6B–D). Furthermore, mRNA assays demonstrated elevated gene expression of macrophage markers (F4/80, CD11b; Fig. 6E, F), inflammatory factors (TNF-α, IL-1β; Fig. 6G, H), and chemokines (MCP-1, CCL2; Fig. 6I, J) in the eWAT of DIO mice. These elevations were attenuated post-treatment, with the BBR + WOG (3:1) combination demonstrating superior efficacy to individual components alone. Similarly, corresponding changes in these inflammatory factors were observed in serum samples (Fig. 6K–M). Concurrently, analysis of post-treatment anti-inflammatory cytokines (IL-10 and TGF-β1) gene expression in adipose tissue revealed that the 3:1 co-treatment group exhibited the most potent effect, significantly restoring the HFD-induced suppression of gene expression (Fig. S4I, J). Treatment significantly reduced serum levels of leptin and resistin compared to DIO controls (Fig. 6N, O), with the BBR + WOG (3:1) combination demonstrating the most pronounced effects. Furthermore, adiponectin, recognised for its insulin-sensitising properties, exhibited decreased levels in HFD-fed mice. These levels were restored following treatment (Fig. 6P). The consistent and robust improvements observed with the BBR + WOG (3:1) combination in DIO mice indicate its synergistic potential in ameliorating adipose inflammation and HFD-induced IR.

### Regulation of lipid metabolism and improvement of liver function in mice by the BBR-WOG combination

**Fig. 7:**
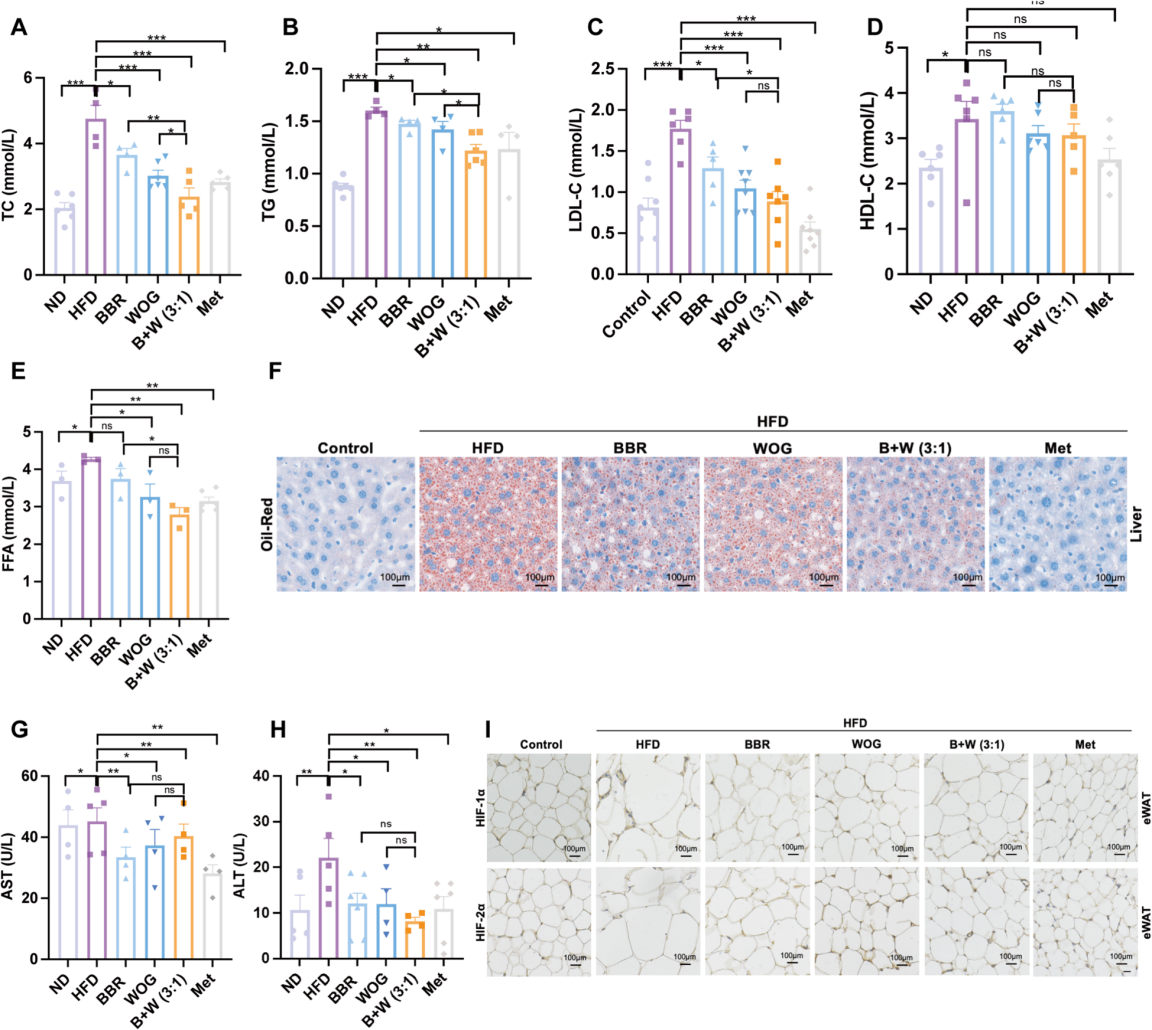
Effects of BBR-WOG combination on lipid metabolism in DIO mice. **A**–**E** Serum levels of TC, TG, LDL-C, HDL-C, and FFA, n ≥ 3. **F** Liver sections were stained with oil red, inverted fluorescence microscope 10×, scale bar 100 μm, n ≥ 3. **G**, **H** Serum AST aspartate aminotransferase and ALT alanine aminotransferase levels in mice, n ≥ 3. **I** Immunohistochemical detection of HIF-1α and HIF-2α protein expression in mouse eWAT, inverted fluorescence microscope 20×, scale

Adipose tissue dysfunction is frequently associated with lipid metabolism disorders. To further evaluate the BBR + WOG (3:1) combination therapy, lipid metabolism parameters were assessed in DIO mice. Initially, elevated serum levels of TC, TG, FFA, HDL, and LDL-C were observed in the HFD group (Fig. 7A–E). The BBR + WOG (3:1) combination demonstrated significantly superior efficacy in reducing serum TC and TG levels compared to the single-agent treatments, indicating a synergistic effect (Fig. 7A, B). Most treatments were effective, with the 3:1 combination group showing particular efficacy, as corroborated by liver immunohistochemical analyses (Fig. 7F). While the BBR + WOG (3:1) combination showed no significant difference from WOG monotherapy in reducing serum FFA and LDL-C, it achieved significantly greater reductions in these parameters than BBR alone at the same dosage (Fig. 7C, E). Meanwhile, serum HDL-C levels showed no significant differences between any treatment group and the HFD group (Fig. 7D). Furthermore, elevated levels of liver function markers AST and ALT in DIO mice were normalised following treatment (Fig. 7G, H), with the combination therapy exhibiting the most pronounced effect. Although the combination’s efficacy in reducing AST levels was not significantly different from that BBR or WOG alone, it achieved a reduction comparable to the most effective groups (BBR and Met) (Fig. 7G). Similarly, the combination administration demonstrated the highest efficacy in reducing ALT levels among all treatment groups (*P* < 0.01) (Fig. 7H). Immunohistochemistry further revealed that the BBR + WOG (3:1) combination reduced HIF-1α expression while increasing HIF-2α expression in eWAT, indicating superior effectiveness compared to either BBR or WOG alone (Fig. 7I). This result also corresponds to the previous investigation of protein changes in vitro (Fig. S5). Consequently, the 3:1 ratio of BBR and WOG appears to synergistically regulate lipid metabolism via the HIF axis, thereby conferring hepatoprotective benefits.

## Discussion

Obesity plays a pivotal role in the pathogenesis of metabolic diseases, with approximately 80–90% of T2DM patients being obese [59]. Clinical evidence indicates that chronic inflammation may serve as a key link between obesity and IR [60, 61]. Notably, hypoxia-inducible factors (HIFs) are crucial regulators of oxygen homeostasis in AT [11]. Couplet medicines, a characteristic form of TCM herb pairing, have been historically employed for disease treatment. Historically, the Coptidis Rhizoma—Scutellariae Radix pair has been extensively utilised in managing obesity and diabetes [62]. Given the documented efficacy of this herb pair in ameliorating T2DM through IR improvement, we hypothesised that its bioactive constituents might synergistically alleviate AT inflammation and treat T2DM via modulation of the HIF signalling pathway. Consequently, based on the established dichotomy of HIF-axis functions under hypoxia conditions, we established a dual-luciferase reporter gene system which initially identified BBR as a HIF-1α inhibitor (Fig. 1). Subsequent investigations identified two bioactive compounds capable of agonizing HIF-2α. Among these, WOG demonstrated notable efficacy in both COCl_2_-induced hypoxia models and normoxic conditions. Notably, WOG significantly suppressed NO production and downregulated the expression of ARG1 (a downstream gene of HIF-2α) (Fig. S1). These findings collectively supported its selection as a potent HIF-2α agonist.

This study employed the Loewe additivity model to determine the optimal compatibility ratio of BBR and WOG by calculating their potency ratio based on respective IC_50_ values [30]. Notably, these findings align with clinical dosing ranges: literature indicates that the clinically effective compatibility ratio of the Coptidis Rhizoma—Scutellariae Radix herb pair typically ranges from 3:2 to 1:3 (crude drug equivalent) [36], corresponding to a BBR:WOG effective dose ratio of approximately 15:1–3:1 [49]. Integrating the potency ratio (≥ 2.3:1) derived from their NO inhibitory effects, we selected three ratios (3:1, 5:1, and 10:1) for optimisation screening. Interaction index analysis demonstrated that only the 3:1 ratio group exhibited a synergistic anti-inflammatory effect across multiple concentration gradients (*τ* < 1) (Fig. 2). In conclusion, by synthesising clinical dosing evidence with synergistic pharmacodynamic evaluation models, this study confirms that the 3:1 BBR:WOG ratio represents the optimal compatibility strategy. This outcome not only provides quantitative guidance for clinical application of the Coptidis Rhizoma—Scutellariae Radix pair, but also establishes an experimental foundation for precision compatibility strategies featuring “toxicity reduction and efficacy enhancement” in TCM formulation.

Macrophages polarisation from the pro-inflammatory M1 phenotype to the anti-inflammatory M2 phenotype represents a tightly regulated process critical to inflammatory homeostasis [63]. This mechanism, involving infiltration into tissue microenvironment, is implicated in diverse inflammatory pathologies [64]. Compelling evidence indicates HIF-1α drives classical M1 activation through phosphofructokinase modulation and inflammatory gene regulation [65], orchestrating metabolic reprogramming and inflammation responses in macrophages [66]. Consequently, hypoxia-induced M1 polarisation propagates inflammation, exacerbating metabolic disorders including obesity and IR. Aligned with this mechanistic framework, we conducted in vitro pharmacological assessments using RAW264.7 cell lines. Cellular experiments findings showed that the 3:1 BBR:WOG combination potently attenuated intracellular inflammation and exerted synergistic anti-inflammation effects in both PA- or LPS-induced models (Figs. 3 and 4). Collectively, these findings validate the 3:1 BBR:WOG ratio as a therapeutic strategy that bridges traditional herbal compatibility with clinical use for treating inflammatory disorders.

To further validate our hypothesis, we evaluated the effects of the BBR-WOG (3:1) combination on IR in DIO mice after 14 weeks of high-fat diet feeding, monitoring key lipid metabolism parameters. In vivo results demonstrated that the combination therapy produced optimal outcomes, comparable to first-line Met. Specifically, treated mice exhibited significant body weight reduction without altered food intake versus HFD controls (Fig. S7A–C), indicating amelioration of HFD-induced obesity independent of caloric restriction. A noteworthy observation is that the combined treatment with BBR and WOG normalised food intake in diet-induced obese (DIO) mice. This effect may stem from the synergistic action of the two compounds, which collectively reduce body weight, restore metabolic balance, and alleviate inflammatory responses in DIO mice. By correcting HFD-activated HIF signalling abnormalities, they normalise appetite and ultimately improve insulin resistance while attenuating inflammation [67–69]. Such synergy likely underlies feeding behaviour normalisation, though the precise mechanism requires further investigation. Metabolic analysis revealed restored respiratory efficiency and significantly enhanced energy expenditure in DIO mice post-combination treatment (Fig. S7). Furthermore, reduced adiposity and improved metabolic indices-including FBG (notable from the 10th week, Fig. S5R), OGTT, and ITT-collectively demonstrated the superior efficacy of the BBR-WOG (3:1) combination over individual agents. These findings were corroborated by biochemical analyses of inflammatory mediators, macrophage markers, and chemokines profiles.

It is established that high-calorie diets constitute a major etiological factor for obesity-induced IR and T2DM. Obesity-driven IR severity correlates with both the total amount and distribution of adipose tissue, wherein expansion of visceral and subcutaneous adipose tissue contributes to impaired insulin resistance [59]. Consequently, we investigated adipose tissue remodelling in DIO mice. Results demonstrated that the 3:1 BBR-WOG combination significantly reduced white adipose tissue mass (Fig. 5E–H). Concurrently, key lipid metabolism parameters-including TC, TG, adiponectin, leptin, etc*.*-were quantified (Fig. 7). While serum HDL-C levels showed an increasing trend compared to the ND group-potentially reflecting proportional changes secondary to HFD-elevated TC/TG/LDL-C (Fig. 7D). All treatment groups (BBR, WOG, and combination) significantly attenuated hepatic lipid accumulation versus HFD controls, with the combination therapy demonstrating superior efficacy. Most significantly, the 3:1 BBR-WOG combination demonstrated concurrent suppression of pro-inflammatory HIF-1α and enhancement of anti-inflammatory HIF-2α expression, as preliminarily validated by immunohistochemical analysis—a finding warranting further mechanistic confirmation (Fig. 7I). Critically, this dual-targeted modulation of HIF isoforms not only validates the synergistic anti-inflammatory mechanism of the Coptidis-Scutellaria herb pair, but also provides an evidence-based framework for optimising clinical applications of TCM in treating obesity-related metabolic disorders through precision compatibility.

Ongoing scholarly debate persists regarding methodologies for assessing pharmacological “synergy” and their non-specific interpretation. Currently, three primary synergy models prevail: the Loewe additive model, the Bliss independent model, and Chow’s neutral effect model [29, 70]. The Loewe additive model-which presumes dose-independent mechanisms without drug interactions-incorporates sigmoidal dose–response relationships and is widely regarded as the “gold standard” for analysing drug interactions since its inception [71]. Consequently, this study employed the Loewe model to quantify synergy by comparing observed combination effects against theoretical additive doses. However, we acknowledge that cross-validation using multiple models was not implemented for BBR-WOG synergy assessment. Future investigations should prioritise establishing robust in vivo screening platforms for optimal ratio determination, while elucidating upstream mechanistic pathways and pharmacokinetic profiles.

In summary, there is growing scientific focus on compatibility-enhanced efficacy in TCM. This study intergartes clinical dosing evidence with mechanistic investigation to identify and validate the 3:1 berberine-wogonin ratio as a dual targeting strategy against HIF-1α/HIF-2α pathways, effectively ameliorating obesity-associated insulin resistance. These finding provide not only a scientific basis for the compatibility theory of the Coptidis-Scutellaria herb pair, but also establish a precision research paradigm bridging dosage, efficacy and targets-paving new avenues for clinical translation of TCM formulation in metabolic disorders.

## Conclusion

In this study, a dual luciferase reporter gene screening system was developed to identify active components from couplet medicines Coptidis Rhizoma-Scutellariae Radix that inhibit HIF-1α and activate HIF-2α. These differential regulatory effects are particularly relevant due to their opposing impacts on hypoxia-triggered inflammation in adipose tissue and IR-associated metabolic disorders. Our findings demonstrate that co-administration of BBR and WOG at a 3:1 ratio synergistically enhances the suppression of inflammatory factors in monocyte-macrophages in vitro, exhibiting efficacy comparable to metformin in suppressing inflammatory responses. In vivo studies further confirmed its potential in attenuating hepatic lipid accumulation, suggesting a synergistic reduction of inflammation in high fat diet-induced adipose tissue and amelioration of IR. Collectively, this study highlights the pharmacological synergy of the Coptidis Rhizoma-Scutellariae Radix couplet, providing a foundation for developing novel therapies for obesity-associated IR.

## Supplementary Information

Additional file1

## Data Availability

No datasets were generated or analysed during the current study.

## Abbreviations

ARG1: Arginase-1
AT: Adipose tissue
BBR: Berberine
WOG: Wogonin
DIO: Diet-induced obese
DM: Diabetes melitus
EE: Energy expenditure
ELISA: Enzyme linked immunosorbent assay
eWAT: White adipose tissue of epididymis
FBG: Fasting blood glucose
FFA: Free fatty acid
HDL-C: High density lipoprotein cholesterol
HFD: High fat diet
HIF: Hypoxia-inducible factor
HRE: Hypoxia-responsive-element
ITT: Insulin tolerance test
iNOS: Inducible nitric oxide synthase
iWAT: Inguinal white adipose tissue
IR: Insulin resistant
IL-1β: Interleukin-1β
IL-6: Interleukin-6
LPS: Lipopolysaccharide
LDL-C: Low density lipoprotein cholesterol
NO: Nitric oxide
OGTT: Oral glucose tolerance test
PA: Palmitic acid
pWAT: Perirenal white adipose tissue
RER: Respiratory exchange rate
sWAT: Subcutaneous white adipose tissue
T2DM: Type 2 diabetes melitus
TNF-α: Tumour necrosis factor-α
TG: Triglyceride
TC: Total cholesterol
WAT: White adipose tissue
TCM: Traditional Chinese Medicine
KO: Knockout
Met: Metformin
