# Supplementary material for: Synergistic potential of Berberine and Wogonin improved adipose inflammation and insulin resistance associated with obesity through HIF-α axis
Source: Chin Med. 2025 Oct 3;20:155. doi: 10.1186/s13020-025-01223-w (PMC12492755; doi:10.1186/s13020-025-01223-w)
Supplement: Supplementary file 1 — Additional file1 [file 13020_2025_1223_MOESM1_ESM.docx]

**Supplementary Figures**

**Fig. S1 Screening of active ingredients with luciferase reporter gene system.** (A) The ARG1-Luc luciferase reporter gene was constructed to screen for HIF-2α agonists under normal (unmodelled) conditions (HEK-293T cells), n=3. RAW264.7 cells were treated with different concentrations of PA for 24 hours, and the cell viability was measured by CCK8. The supernatant was taken to detect the NO level. (B) Cell viability after PA intervention at different concentrations, n≥6. (C) NO level after PA treatment, n≥6. (D, E) PA, LPS-induced effects of different drugs on NO levels, n≥6. (F, G) PA, LPS-induced effects of different drugs on ARG1 mRNA levels, n≥3. (***P<0.001, **P<0.01, *P<0.05, *ns* not significantly different)

**Fig. S2 Effects of different concentrations of BBR and WOG on PA-induced proliferation of RAW 264.7 cells and gene expression of ARG1 and iNOS.** RAW264.7 cells were treated with 100 μM PA and different concentrations of BBR or WOG for 24 hours. (A, B) CCK8 was used to test cell viability, n≥6. The gene expression was detected by qPCR. (C, D) iNOS gene expression and ARG1 gene expression, n≥3. (E, F) NO concentration was measured to determine the IC_50_ value, n≥6. (***P<0.001, **P<0.01, *P<0.05, *ns* not significantly different)

**Fig. S3 Effects of BBR and WOG on gene expression of inflammatory cytokines in macrophages.** Induced by 100 μM PA and 1 μg/mL LPS, and different concentrations of BBR and WOG on RAW 264.7 were treated for 24 hours or 12 hours. The gene expression was detected by qPCR. (A-F) Under PA and LPS modelling, the effects of BBR administration at concentration gradients of 1.25, 2.5, and 5 μM on the gene expression of IL-6, IL-1β, and TNF-α, n≥3. (G-L) Under PA and LPS modelling, the effects of WOG administration at concentration gradients of 1.25, 2.5, and 5 μM on the gene expression of IL-6, IL-1β, and TNF-α, n≥3. (***P<0.001, **P<0.01, *P<0.05, *ns* not significantly different)

**Fig. S4 Effects of BBR and WOG on anti-inflammatory factors.** RAW 264.7 was induced with 100 μM PA and 1 μg/mL LPS, and treated with BBR, WOG at 5 μM, or BBR: WOG 3:1 (3.75:1.25) for 24 or 12 hours. ELISA detected cytokine secretion in cell supernatant. Gene expression was detected by qPCR. (A-D) The effects of BBR and WOG, respectively and in combination, on the mRNA expression of IL-10 and TGF-β1 under PA and LPS modelling conditions, n≥3. (E-H) The effects of BBR and WOG on the secretion of inflammatory factors IL-10 and TGF-β1, respectively and in combination, under PA and LPS modelling conditions, n≥3. (I, J) Effects of administration on IL-10 and TGF-β1 mRNA gene expression in eWAT adipose tissue, n≥3. (***P<0.001, **P<0.01, *P<0.05, *ns* not significantly different)

**Fig. S5 Effects of BBR and WOG on the expression of adipose inflammatory proteins and genes based on the HIF axis.** 3T3-L1 cells were induced with 100 μM PA and 1 μg/mL LPS, and treated with BBR, WOG at 5 μM, or BBR:WOG 3:1 (3.75:1.25) for 24 or 12 hours. Protein expressions were detected by Western Blot, while gene expressions were detected by PCR. (A, B) Under PA or LPS model, the effects of HIF-1α protein expressions of BBR, WOG and BBR:WOG (3:1) administration, n=3. (C, D) Under PA or LPS model, the effects of iNOS protein expressions of BBR, WOG and BBR:WOG (3:1) administration. (E-H) Statistics of the quantity of HIF-1α or iNOS protein expression levels, n=3. (I, J) Under PA or LPS model, the effects of HIF-2α protein expressions of BBR, WOG and BBR:WOG (3:1) administration, n=3. (K, L) Under PA or LPS model, the effects of ARG1 protein expressions of BBR, WOG and BBR:WOG (3:1) administration, n=3. (M-P) Statistics of the quantity of HIF-2α or ARG1 protein expression levels, n=3. (Q-T) The effects of BBR and WOG intervention on the gene expression of HIF-1α or iNOS after PA or LPS modelling, n=3. (U-X) The effects of BBR and WOG intervention on the gene expression of HIF-2α or ARG1 after PA or LPS modelling, n=3. (***P<0.001, **P<0.01, *P<0.05, *ns* not significantly different)

**Fig. S6 *In vivo* acute toxicity test.** (A) Schematic diagram of acute toxicity test in mice. (B) Comparison of hair color and body posture in mice after oral administration of 400 mg/kg for one week. (C) Statistics of the proportion of each organ to body weight (g/g), n≥6. (D) Comparison of organ appearance between ND group and treatment group (400 mg/kg), n≥6. (E) HE stained sections of ND group and treatment group (400 mg/kg), 4 ×, 500 μm, n≥6. (***P<0.001, **P<0.01, *P<0.05, *ns* not significantly different)

**Fig. S7 Effects of BBR-WOG combination on body weight, feeding and basal metabolism in DIO mice.** High-fat diet mice were administered for 14 weeks. (A) Mouse body weight-time relationship curves, n≥6. (B) Mouse food intake-time-variation curves, n≥8. (C) Average weekly food intake of mice, n≥8. (D, E) 24-hour oxygen consumption, n=3. (F, G) 24-hour carbon dioxide release, n=3. (H-J) 24-hour respiratory exchange rate (RER), n=3. (K-M) 24-hour energy expenditure (EE) in mice, n=3. (N) eWAT weight/body weight ratio, n≥6. (O) Body fat percentage, i.e., total body fat weight/body weight, n≥6. (P) 10-week insulin tolerance curve in mice on a high-fat diet, n≥5. (Q) Oral glucose tolerance curve in mice on a high-fat diet for 10 weeks, n≥5. (R) Fasting blood glucose in mice on a high-fat diet for 10 weeks, n≥5. (***P<0.001, **P<0.01, *P<0.05, *ns* not significantly different)
